# Upregulation of ATP Binding Cassette Subfamily C Member 5 facilitates Prostate Cancer progression and Enzalutamide resistance via the CDK1-mediated AR Ser81 Phosphorylation Pathway

**DOI:** 10.7150/ijbs.59559

**Published:** 2021-04-12

**Authors:** Guangjie Ji, Shiming He, Cong Huang, Yanqing Gong, Xuesong Li, Liqun Zhou

**Affiliations:** Institute of Urology, Peking University. Department of Urology, Peking University First Hospital. National Urological Cancer Center of China, Beijing, China.

**Keywords:** Castration-resistant prostate cancer, ABCC5, CDK1, AR, enzalutamide

## Abstract

The treatment of advanced prostate cancer, castration-resistant prostate cancer, remains challenging. The mechanisms of action of ATP binding cassette subfamily C member 5 (ABCC5) in prostate cancer and its relationship with drug resistance are still unclear. Expression and prognostic analyses of ABCC5 were performed through bioinformatic methods and immunohistochemistry analyses in multiple public databases as well as in our own prostate cancer cohort. The biological function of ABCC5 in prostate cancer cells was evaluated by *in vitro* and *in vivo* cell proliferation and migration and invasion assays. The regulation of CDK1 by ABCC5 was determined via RT-qPCR, western blots, and immunofluorescence. ABCC5 was significantly overexpressed in prostate cancer and positively associated with unfavorable clinicopathological features and prognosis. Upregulation of ABCC5 could enhance the cell proliferation, migration, and invasion of prostate cancer *in vitro* and *in vivo*. Mechanistically, ABCC5 exerts a protumor effect by binding to and inhibiting the protein degradation of CDK1, which promotes the phosphorylation of AR at Ser81 by CDK1 and activates the transcriptional activity of AR on target genes. Moreover, the addition of a CDK1 inhibitor or knockdown of CDK1 significantly improved the efficacy of enzalutamide on prostate cancer cells. The ABCC5-CDK1-AR regulatory pathway could be a potential therapeutic target for advanced prostate cancer, especially castration-resistant prostate cancer (CRPC), to enhance the therapeutic effect of enzalutamide.

## Introduction

Prostate cancer has become one of the most prevalent malignancies affecting the quality of life of older men worldwide 1, 2. Although there are a variety of treatments for prostate cancer, especially early-stage prostate cancer, effective treatments for advanced prostate cancer are lacking 3-6. As a type of advanced prostate cancer with a poor prognosis, metastatic castration-resistant prostate cancer (mCRPC) has attracted increasing attention from scholars and physicians 7, 8. Particularly for patients with metastatic and castration-resistant prostate cancer, combination regimens including androgen deprivation therapy and radiotherapy and chemotherapy are not sufficiently effective 9-15. The search for new and effective drug targets is important for improving the efficacy of prostate cancer treatment.

ATP binding cassette subfamily C member 5 (ABCC5), also called multidrug resistance-associated protein 5, has been reported to play an essential cancer-promoting role in various tumors 16-19. Current studies on ABCC5 have mainly focused on the impact of its transmembrane protein transport function on the efficacy of cancer chemotherapy. ABCC5 (rs9823696) was identified as a new specific risk locus associated with esophageal adenocarcinoma 20. Inhibition of the FOXM1-ABCC5 axis could increase the intracellular concentrations of paclitaxel and overcome paclitaxel chemoresistance in nasopharyngeal carcinoma 16. Additionally, ABCC5 is downregulated by several microRNAs in multiple types of cancer 17, 21-23. However, the concrete biological mechanisms of ABCC5 in tumor progression and drug resistance remain unclear, especially the downstream regulatory pathway of ABCC5.

In this study, we focused on the specific regulatory mechanism of ABCC5 in prostate cancer progression. We first demonstrated that ABCC5 was overexpressed in prostate cancer and that a high expression level of ABCC5 resulted in aggressive clinicopathologic features and an unfavorable prognosis. ABCC5 promotes the proliferation, migration, and invasion of prostate cancer tumor cells *in vitro* and *in vivo*. Through bioinformatics analysis, we found that the possible downstream molecule of ABCC5 is CDK1 and experimentally verified that ABCC5 inhibits the degradation of CDK1 through its binding to CDK1, which in turn activates the downstream AR signaling pathway to exert protumor effects. Moreover, the addition of RO-3306, an inhibitor of CDK1, significantly inhibited tumor growth and enhanced enzalutamide sensitivity, providing a potential therapeutic target for clinical treatments.

## Materials

### Bioinformatic data collection and mining

To comprehensively evaluate the expression level of ABCC5 in prostate cancer samples, we downloaded the transcriptome data and clinical information of prostate cancer cohorts in The Cancer Genome Atlas (TCGA, https://portal.gdc.cancer.gov/) and Gene Expression Omnibus (GEO, https://www.ncbi.nlm.nih.gov/geo/). The relationship between ABCC5 expression and immune cell infiltration in prostate cancer was explored in the TIMER (Tumor Immune Estimation Resource) website (http://timer.cistrome.org/) 24-26.

### Weighted-gene correlation network analysis (WGCNA)

Weighted-gene correlation network analysis is a systems biology method that uses gene expression data to build a scale-free network 27. First, we extracted the expression data of TCGA-PRAD samples to implement WGCNA. All the genes expressing NA according to the expression profile were removed. Two outlier samples were removed based on the clustering distance after calculating each gene's variance per sample and selecting genes with a standard deviation higher than zero. Next, we used the R software package WGCNA to construct a weighted coexpression network. To ensure that the network is a scale-free network, we chose a soft threshold of β = 6. We converted the expression matrix into the adjacency matrix and then transformed the adjacency matrix into a topological matrix (TOM). Based on TOM, we used the average-linkage hierarchical clustering method to cluster genes according to the standard of the mixed dynamic shear tree and set the minimum number of genes in network modules to 30. After using the dynamic shear method to determine the gene modules, we calculated the feature vector values (eigengenes) of each module in turn and then performed cluster analysis on the modules, merging the modules that were closer to each other into a new module and setting the height to 0.25. A total of 18 modules were obtained, where the gray module is a collection of genes that cannot be aggregated into other modules. According to the feature vectors of each module, we calculated the gene trait significance (GtS) between these modules and ABCC5 expression to measure the degree of relation between the genes and scores. GtS = 0 indicates that the genes are not related to the phenotype.

### Patient selection

We collected clinical information and formalin-fixed paraffin-embedded samples from a total of 149 prostate cancer patients who underwent radical prostatectomy without preoperative therapy at Peking University First Hospital from January 2012 to December 2014. Our study was approved by the Biomedical Research Ethics Committee of Peking University First Hospital. All patients in this study signed an informed consent form.

### Cell culture and transfection

The C4-2 and VCaP cell lines were obtained from the Institute of Cell Research, Chinese Academy of Science, Shanghai, China. Cell lines were cultured in RPMI 1640 (Corning, USA) medium supplemented with 10% fetal bovine serum (FBS) in 37 °C incubators with 5% CO_2_. We designed shRNA sequences for ABCC5 and CDK1 and then inserted them into the pLKO.1 vector. The details of the shRNA sequences for ABCC5 and CDK1 are shown in Supplementary Table S1. The coding DNA sequence of ABCC5 was produced by PCR and inserted into the pLVX vector with an N-terminal S-tag label. Lentivirus packaging was completed in HEK-293T cells with a three-vector system of interest vector, viral packaging vector (psPAX2), and viral envelope vector (pMD2G) at a 4:3:1 ratio. Finally, prostate cancer cells were infected with lentivirus and selected with puromycin (2 μg/mL) and hygromycin (10 μg/mL).

### Generation of enzalutamide-resistant (ENZ-R) cell lines

ENZ-R cells were generated by culturing C4-2 and 22Rv1 cells under 10 μM enzalutamide for six months before the experiment. After generation, ENZ-R cells were maintained in media with 10 μM enzalutamide.

### RNA extraction and real-time quantitative PCR

Total RNA was extracted from prostate cancer cells by using TRIzol reagent (Invitrogen; Thermo Fisher Scientific, Inc.) and reverse-transcribed with an RT-PCR kit (TransGENE, Beijing, China). Quantitative PCR was conducted in the ABI Prism 7000 fluorescent quantitative PCR system (Applied Biosystems, Foster City, CA, USA) according to the manufacturer's instructions. The expression level was normalized to that of TUBA, and the sequences of all the primers in this study are shown in Supplementary Table S2.

### Immunohistochemistry (IHC)

All paraffin-embedded tissues were cut into 5 μm sections and placed on slides. After the paraffin wax was removed, the tissue was brought to a boil in 10 mM sodium citrate buffer, pH 6.0, for ten minutes for antigen unmasking. Then, 3% H2O2 was added to the surface of the tissues to inactivate endogenous peroxidases for 20 minutes. Subsequently, 10% goat serum was used to reduce nonspecific binding for 1 hour at room temperature. The tissue was incubated with anti-ABCC5 or anti-CDK1 primary antibody at 4°C overnight and then processed with staining reagent (PV-9000, ZSGB-Bio, China) according to the manufacturer's instructions. Each slide was scored as negative, weak, moderate, and strong based on the staining frequency and intensity by two independent pathologists.

### Western blot analysis

Total cellular protein was extracted using 1% NP-40 containing 1 mM phenylmethylsulfonyl fluoride (PMSF) and quantified through a bicinchoninic acid assay (Sigma, USA). The protein samples were separated in SDS-PAGE gels and transferred to polyvinylidene difluoride membranes. The protein bands were visualized with an ImmobilonTM Western kit (Millipore, Billerica, MA) under the Syngene G: BOX imaging system (Frederick, USA). All information about the primary antibodies in this study is shown in Supplementary Table S3.

### Immunofluorescence

Cancer cells were placed on glass slides for 24 hours before the experiment. The cells were fixed with 4% paraformaldehyde, washed with PBS for 15 minutes, and then permeabilized with 0.5% Triton X-100. Subsequently, the cells were incubated with the primary antibodies at room temperature for 2 hours.

### Immunoprecipitation

After treatment with 10 μM MG132 for 24 h, cellular protein was extracted with 1% NP-40 and 1 mM PMSF and quantified using a bicinchoninic acid assay. S-tagged beads or target primary antibody were added to whole cell lysates and incubated at 4 °C overnight. After washing and discarding the beads, we collected the supernatant liquid for further immunoblot analyses.

### S-tag pulldown assay and mass spectrometry analysis

Protein lysates were prepared by homogenizing the cells in 1% NP40 buffer containing PMSF and a proteinase inhibitor cocktail (Roche). Protein lysates were incubated with S-tagged beads (Novagen) at 4 °C for 2 h. After centrifugation and three washes with 1% NP-40 buffer, the precipitated complex was boiled with protein loading buffer and separated by SDS-PAGE. The gel was stained with a silver stain kit (Beyotime). The precipitated complex was then analyzed by mass spectrometry. Mass spectrometry analysis was performed using an AB SCIEX MALDI TOF-TOF 5800 Analyzer.

### Wound healing assay

After seeding the prostate cancer cells in 6-well plates for 24 hours, the cells were scratched with a 200 μl sterile pipette. The wound was photographed with a microscope (Leica DM IL, Leica Microsystems, Germany) equipped with a digital camera (Leica DFC300FX) 24, 48, 72, and 96 hours later.

### MTS cell proliferation assay

A total of 2000 cells/well were seeded in a 96-well plate and cultured for 6 days. During this time period, cell proliferation was measured every day. After addition of 20% MTS reagent (Abcam, USA) diluted in PBS and incubation for 4 hours in standard culture conditions, we measured the absorbance at 490 nm to evaluate the cell proliferation.

### Colony formation assay

A total of 1000 cells/well were seeded in a 6-well plate and cultured for 2 weeks. Then, after discarding the medium, we fixed and stained the cancer cells with 0.5% crystal violet methanol solution for 1 hour. The samples were dried by airing, and the 6-well plates were scanned.

### Transwell migration and invasion assay

In the Transwell migration assay, 10000 cells were plated in the upper chamber of a Transwell apparatus (24-well insert, 8 μm pore size, Corning) containing 200 μl of serum-free RPMI-1640. The lower chamber was filled with 700 μL of RPMI-1640 containing 10% fetal bovine serum. After 24 hours, the adherent cells on the underside were stained with 0.5% crystal violet in methanol for 30 minutes.

In the Transwell Matrigel invasion assay, 1000 cells in a Transwell apparatus (24-well insert, 8 μm pore size, Corning) were coated with Matrigel (diluted 1:8 with PBS, product number 354234, Corning, Inc., NY, USA) for the invasion assay. The culture conditions were the same as those described in the Transwell migration assay. After 24 hours, the adherent cells on the underside were stained with 0.5% crystal violet in methanol for 30 minutes. The cells on the underside were photographed with a microscope (Leica DM IL, Leica Microsystems) equipped with a digital camera (Leica DFC300FX) and counted.

### *In vivo* tumorigenicity assay

A total of 1 × 10^7^ VCaP cells with upregulated ABCC5 levels were subcutaneously injected into the flanks of 4 week-old male BALB/c immunodeficient nude mice (eight mice per group). Tumor volumes were measured once every week for 4 weeks. Then, the tumors were removed, and their sizes and weights were determined. The animal study was approved by the Institutional Animal Experiment Committee of Peking University First Hospital.

### Statistical analyses

All statistical tests were performed using R version 3.6, including Student's t test, the chi-square test, and the nonparametric test. The results are shown as the mean ± SEM. The Kaplan-Meier method with the log-rank test was applied to calculate survival benefits. Differences with p less than 0.05 were considered statistically significant (*p < 0.05, **p < 0.01 and ***p < 0.001).

## Results

### ABCC5 is overexpressed in prostate cancer

**Figure 1:**
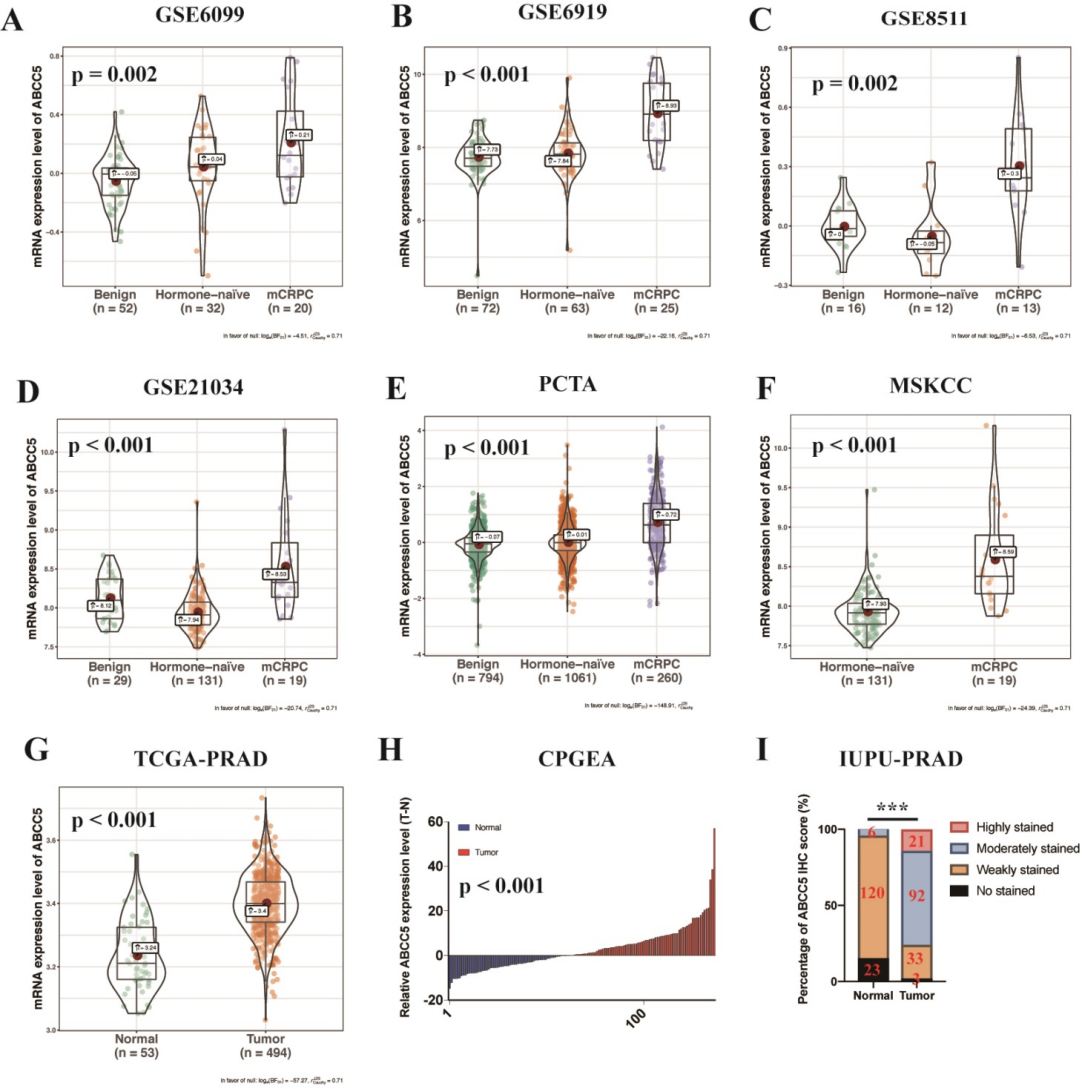
** The expression of ABCC5 is upregulated in prostate cancer from different datasets.** A. GSE6099, B. GSE6919, C. GSE8511, D. GSE21034, E. PCTA, F. MSKCC, G. TCGA-PRAD, H. CPGEA, I. IUPU-PRAD. PCTA, prostate cancer transcriptome atlas. MSKCC, Memorial Sloan Kettering Cancer Center prostate cancer cohort. TCGA-PRAD, The Cancer Genome Alta Prostate Adenocarcinoma. CPGEA, Chinese Prostate Cancer Genome and Epigenome Atlas. IUPU-PRAD, Institute of Urology, Peking University, Prostate Adenocarcinoma. ***P < 0.001.

To fully and comprehensively explore the expression of ABCC5 in prostate cancer, RNA-seq data of prostate samples from TCGA, GEO, Memorial Sloan Kettering Cancer Center (MSKCC), and Chinese Prostate Cancer Genome and Epigenome Atlas (CPGEA) were analyzed. We analyzed the expression profiles of different prostate tissues from several databases and found that the expression levels of ABCC5 showed an obvious progressive increase in metastatic castration-resistant prostate cancer (mCRPC) compared with benign hyperplastic tissues and primary tumor foci (Figure 1A-H, p ≤ 0.002). Moreover, ABCC5 mRNA expression was higher in prostate cancer tissues than in matched adjacent normal prostate tissues, as demonstrated in the CPGEA dataset (Figure 1I). Furthermore, to evaluate the expression of ABCC5 at the protein level, we analyzed a total of 149 prostate cancer samples from the Institute of Urology, Peking University prostate cancer dataset (IUPU-PRAD) by immunohistochemistry. Consistent with the transcriptome results, we also found that ABCC5 was strongly overexpressed in the tumor tissue at the protein level compared with normal prostate tissue (Figure 1I, Supplementary Figure 1).

### Upregulated ABCC5 correlates with poor prognosis

**Figure 2:**
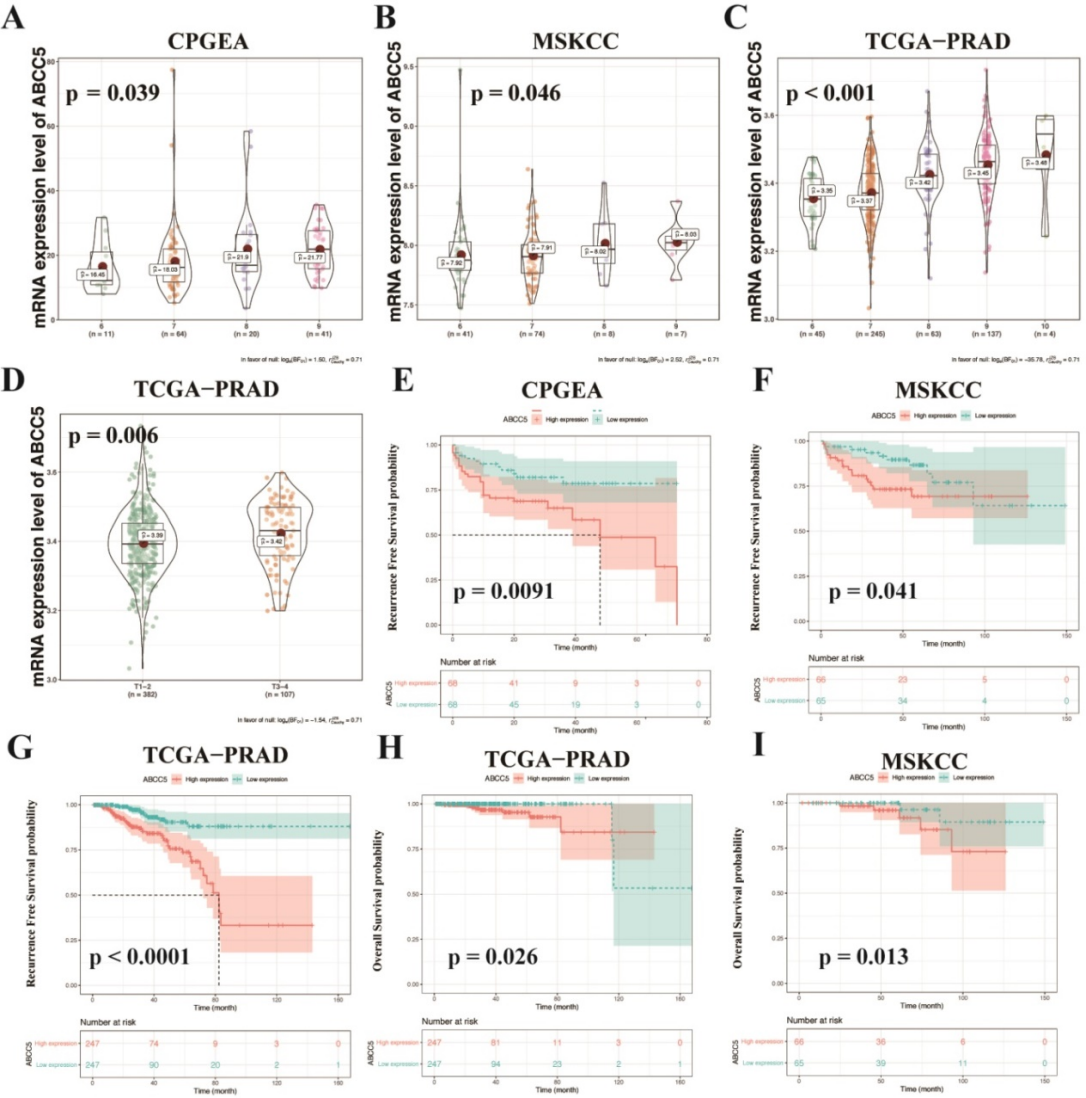
** ABCC5 expression is associated with pathological features and prognosis of prostate cancer.** A-C. Association of ABCC5 expression with different GS of prostate cancer in CPGEA (A), MSKCC (B), PCGA-PRAD (C). D. Association of ABCC5 expression with different pathological stages of prostate cancer in TCGA-PRAD. E-G. Recurrence survival curves of ABCC5 high and low expression groups in CPGEA (E), MSKCC (F), PCGA-PRAD (G). H-I. Overall survival curves of ABCC5 high and low expression groups in TCGA-PRAD (H), MSKCC (I). CPGEA, Chinese Prostate Cancer Genome and Epigenome Atlas. MSKCC, Memorial Sloan Kettering Cancer Center prostate cancer cohort. TCGA-PRAD, The Cancer Genome Alta Prostate Adenocarcinoma.

To further explore the relationship between ABCC5 overexpression and clinicopathological characteristics, we compared ABCC5 expression in different pathological scores and tumor stages. As shown in Figure 2, we found that the expression level of ABCC5 was significantly higher in prostate cancer patients with higher GS scores from the CPGEA cohort (p = 0.039, Figure 2A), MSKCC cohort (p = 0.046, Figure 2B), and TCGA-PRAD cohort (p < 0.001, Figure 2C) than those with low scores. Similarly, ABCC5 expression was also highly associated with tumor stage (p = 0.006, Figure 2D). Next, we evaluated the relationship between ABCC5 expression levels and the prognosis of prostate cancer patients in several prostate cancer cohorts, including CPGEA, MSKCC, and TCGA-PRAD. In CPGEA (p = 0.0091), MSKCC (p = 0.041), and TCGA-PRAD (p < 0.0001), we found shorter progression-free survival (RFS) times in prostate cancer patients with high ABCC5 expression than those with low expression (Figure 2E-G). Similarly, in TCGA-PRAD (p = 0.026) and MSKCC (p = 0.013), prostate cancer patients with high ABCC5 expression also had shorter overall survival times than those with low ABCC5 expression (Figure 2H-I). Furthermore, in multivariable Cox regression analysis (Table 2), we found that the expression of ABCC5 is independent predictor of RFS in TCGA-PRAD (p = 0.025).

### ABCC5 facilitates the proliferation, migration and invasion of prostate cancer cells

**Figure 3:**
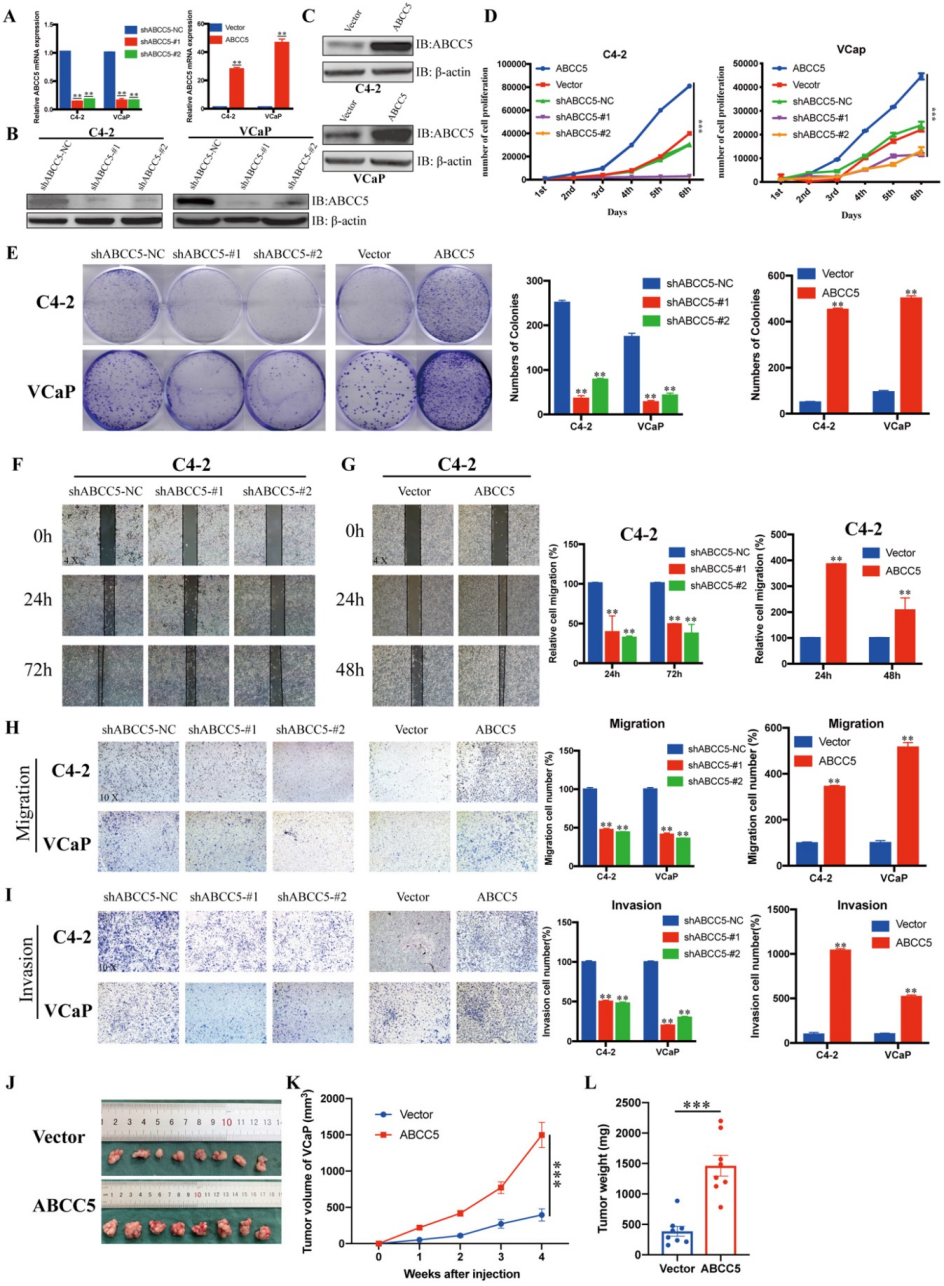
** ABCC5 promotes the malignant progression of prostate cancer *in vivo* and *in vitro*.** A-C. RT-qPCR and western blot analyses of prostate cancer cells infected with a lentivirus-mediated ABCC5-overexpressing vector or ABCC5 shRNAs. D. MTS cell proliferation assay of prostate cancer cells infected with a lentivirus-mediated ABCC5-overexpressing vector or ABCC5 shRNAs. E. Colony formation assay, Representative images of clone formation of shABCC5, ABCC5 and control group in C4-2 and VCaP cells (left). Quantification of colonies by the described cells (right). F-G. Wound healing assay, Representative images of wound-induced cell migration by the C4-2-shABCC5 (F), C4-2-ABCC5 (G) and control cells (4x, left). Quantification of migration by the described cells (right). H. Transwell migration assay, Representative images of transwell migration assay of ABCC5-knockdown and ABCC5-overexpressed and control cells (10x, left). Quantification of transwell migration by the described cells (right). I. Transwell invasion assay, Representative images of transwell invasion assay of ABCC5-knockdown and ABCC5-overexpressed and control cells (10x, left). Quantification of transwell invasion by the described cells (right). J-L. Subcutaneous tumorigenesis experiment in nude mice. Representative images of subcutaneous tumor of nude mice injected with ABCC5-overexpressed and control cells. Eight mice in each group (J). Growth curve of subcutaneous tumor volume (K). Comparison of tumor weight at 4^th^ week between control and ABCC5-overexpressed groups (L). **P < 0.01, ***P < 0.001.

To further explore the effect of ABCC5 on prostate cancer, we knocked down and overexpressed ABCC5 in two prostate cancer cell lines, C4-2 and VCaP, respectively (Figure 3A-C), and performed a series of tumor cell functional assays. The results of MTS and clone formation assays showed that the proliferation of prostate cancer cells was significantly inhibited and enhanced by ABCC5 knockdown and overexpression, respectively (Figure 3D-E). Subsequently, we examined the effect of ABCC5 on the migration of prostate cancer cells, and the results of scratch and transwell migration assays showed that knockdown of ABCC5 significantly reduced the migration of prostate cancer cells, while overexpression of ABCC5 significantly enhanced the migration of prostate cancer cells (Figure 3F-H, Supplementary Figure 2A-B). Similarly, the invasive ability of prostate cancer cells was suppressed with the downregulation of ABCC5 expression and enhanced with the increase in ABCC5 expression (Figure 3I). The procarcinogenic role of ABCC5 in prostate cancer was also confirmed by the results of the rescue assay (Supplementary Figure 2C-D). To further validate the effect of ABCC5 on prostate cancer in animals, we chose to perform subcutaneous tumorigenesis experiments using ABCC5-overexpressing VCaP cells injected into immunodeficient nude mice. We found that high expression of ABCC5 significantly accelerated the tumor growth of prostate cancer (Figure 3J).

### Downstream pathway prediction of ABCC5 combined with bioinformatics analysis

**Figure 4:**
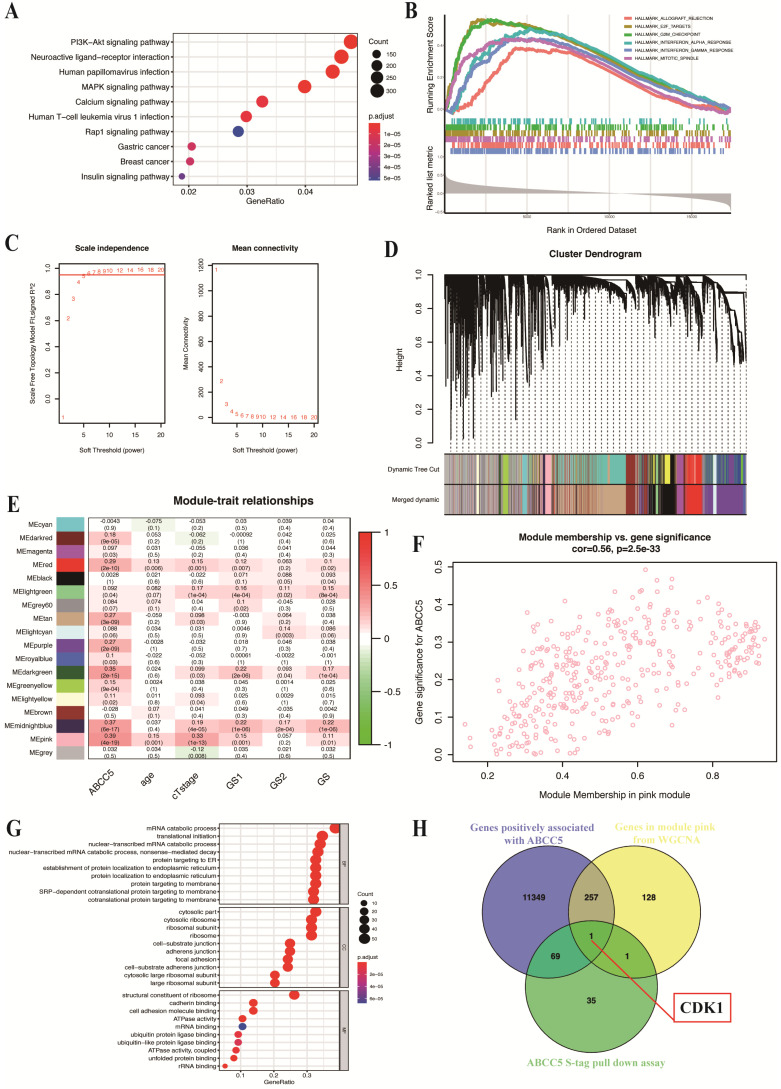
** CDK1 may be the potential molecular in the downstream of ABCC5 in prostate cancer based on bioinformatic analyses.** A. GO analysis of genes positively associated with ABCC5 in TCGA-PRAD. B. GSEA plot of genes positively associated with ABCC5 based on hallmarks gene sets. C. Optimizing soft threshold to make sure there is a scale-free network. D. cluster dendrogram of module construction. E. correlation between modules and ABCC5 expression and other clinicopathological features of prostate cancer. F. correlation between gene significance in module pink and ABCC5 expression. G. GO analysis of proteins in ABCC5 S-tag pull-down assay. H. The intersection of genes positively associated with ABCC5, genes in module pink from WGCNA, and proteins of ABCC5 S-tag pull-down assay. GO, gene ontology. TCGA-PRAD, The Cancer Genome Alta Prostate Adenocarcinoma. WGCNA, Weighted-gene correlation network analysis.

To explore the specific mechanism of the cancer-promoting effect of ABCC5 in prostate cancer, we divided the prostate cancer patients in the TCGA-PRAD cohort into an ABCC5 high expression group and an ABCC5 low expression group according to the expression level of ABCC5. By comparing the differential expression of all genes in these two groups, we obtained a total of 11,676 genes with upregulated expression in the ABCC5 high expression group compared to the ABCC5 low expression group. We performed GO analysis of downstream pathways and GSEA of these differentially expressed genes and found that ABCC5 highly expressed in prostate cancer may act through multiple tumor-related pathways, including the PI3K-Akt and MAPK/ERK signaling pathway and E2F target genes (Figure 4A-B). Previous studies have reported a regulatory relationship between the PI3K-Akt and MAPK/ERK signaling pathways and CDK1 28, 29. Immediately thereafter, we analyzed transcriptomic data from prostate cancer patients using the WGCNA approach and found that the pink module was highly correlated with ABCC5 expression levels (R = 0.39, p = 4e-19) (Figure 4C-E). Moreover, 387 genes in the pink module were significantly correlated with ABCC5 (Figure 4F). Using protein pulldown and mass spectrometry techniques, we then searched for proteins that might bind to ABCC5 and in this way mediate the oncogenic effects of ABCC5 at the protein level. We identified 69 proteins that may bind to ABCC5 in the cell lines overexpressing ABCC5 compared to the controls, focusing on biochemical processes such as protein membrane localization and molecular functions such as ubiquitinated protein binding (Figure 4G). Finally, we assessed the intersection of the set of differentially expressed genes positively associated with ABCC5 expression, the full set of genes in the pink module that are highly associated with ABCC5, and the proteins identified by protein profiling that may interact with ABCC5 and identified a potential downstream molecule, CDK1, that may interact with ABCC5 (Figure 4H).

### ABCC5 binds CDK1and stabilizes the protein levels

**Figure 5:**
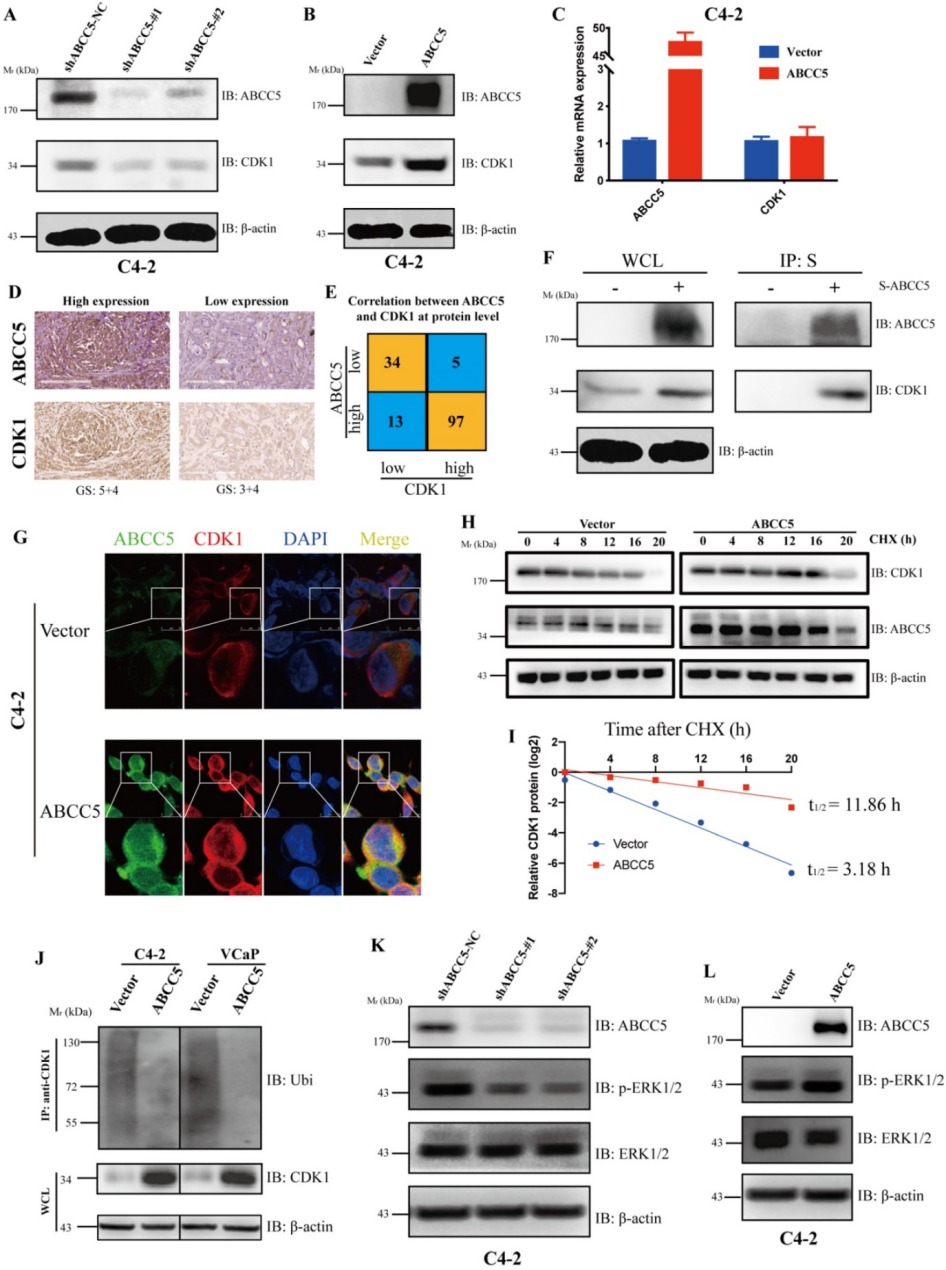
** ABCC5 upregulates CDK1 protein expression by binding to and inhibiting the ubiquitinated degradation of CDK1.** A. Knockdown of ABCC5 reduced the protein expression of CDK1 in the prostate cancer cell. B. Overexpressed ABCC5 increased the protein expression of CDK1 in the prostate cancer cell. C. The mRNA expression of CDK1 in ABCC5-overexpressed and control cells. D. Representative images of ABCC5 and CDK1 protein expression in IUPU-PRAD cohort by IHC. Scale bar 200 µm. E. Correlation between ABCC5 and CDK1 at the protein level. F. The result of immunoprecipitation revealed that ABCC5 binds to CDK1 at the protein level (p < 0.01). G. ABCC5 and CDK1 were co-localized by immunofluorescence. H-I. ABCC5 prolongs the half-life of CDK1. Control and ABCC5 overexpressing prostate cancer cells were treated with Cycloheximide (CHX) (50 µM), and then the protein levels of CDK1 were detected at different time periods. J. ABCC5 inhibits the ubiquitin proteasome-dependent degradation pathway of CDK1. Control and ABCC5 overexpressing prostate cancer cells were treated with the proteasome inhibitor MG132 (10 µM) for 24 hours, and then ubiquitinated CDK1 expression was examined. K-L. Knockdown and overexpression of ABCC5 can inhibit and activate the p-ERK1/2 pathway in prostate cancer cells, respectively.

To further explore the regulatory relationship between ABCC5 and CDK1, we examined the mRNA and protein expression levels of CDK1 in the ABCC5 knockdown cell lines and the ABCC5 overexpression cell lines. We found that ABCC5 could stabilize the protein level of CDK1 without affecting mRNA expression (Figure 5A-C). Further, we proposed that ABCC5 could specifically bind and stabilize the protein level of CDK1. To fully test our hypothesis, we examined the protein expression levels of ABCC5 and CDK1 in our own prostate cancer cohort and revealed that the protein expression of CDK1 was highly positively correlated with the protein expression of ABCC5 (Figure 5D-E). Immediately afterward, we also confirmed that ABCC5 could bind to CDK1 using IP (Figure 5F, Supplementary Figure 3A). By laser confocal immunofluorescence, we verified that ABCC5 could indeed bind and upregulate CDK1; thus, does ABCC5 stabilize the protein level of CDK1 by inhibiting its protein degradation? We found that the half-life of CDK1 was prolonged and that ubiquitinated CDK1 was reduced after overexpression of ABCC5 (Figure 5H-J), suggesting that ABCC5 binds to CDK1 and inhibits the ubiquitination of CDK1, thus stabilizing the CDK1 protein levels. We also found that ABCC5 could activate the ERK1/2 and Akt signaling pathway (Figure 5K-L, Supplementary 3B, C), which confirmed the results of the previous signal pathway analyses.

### Inhibition of CDK1 blocked the cancer-promoting effect of ABCC5

**Figure 6:**
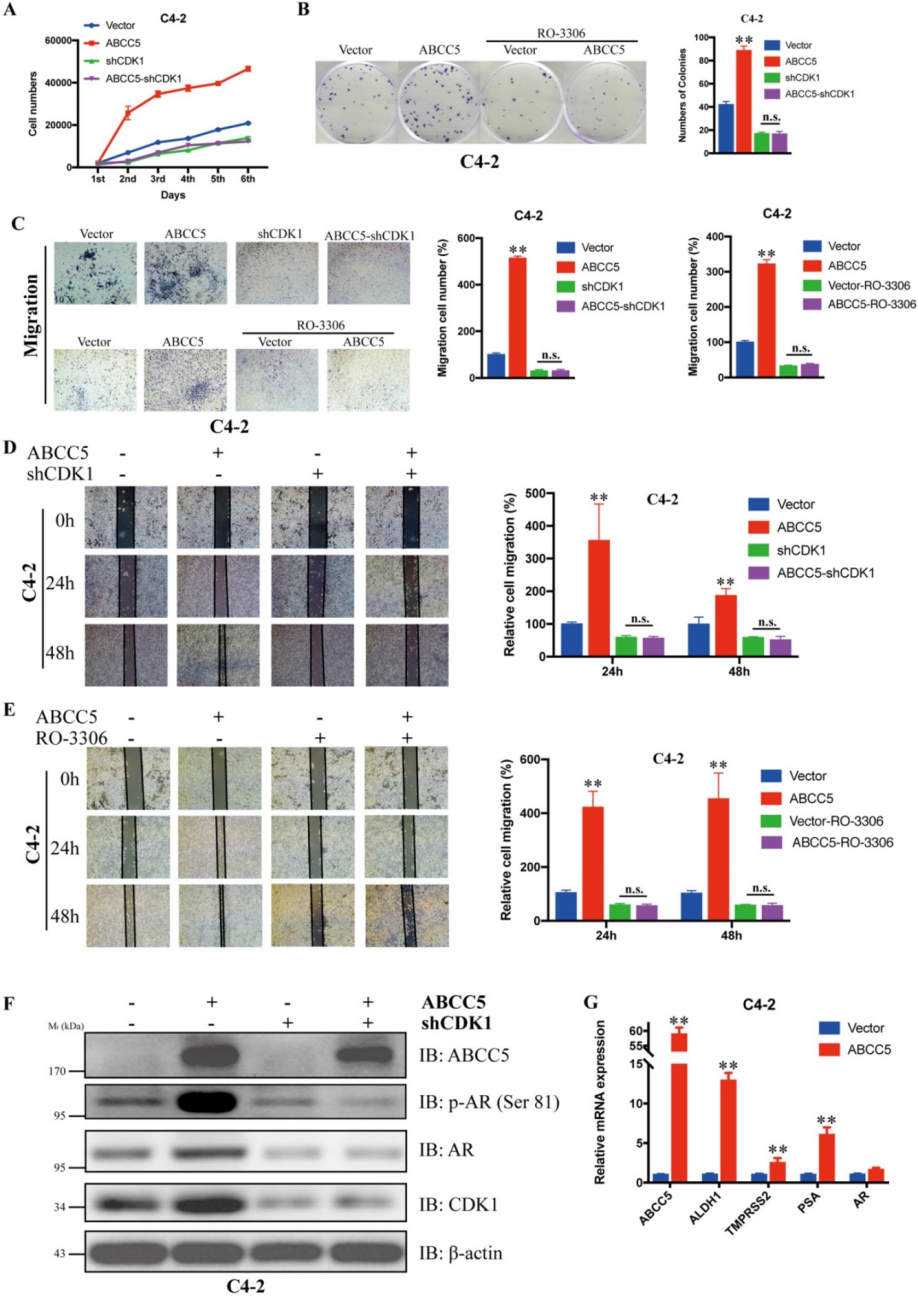
** CDK1 mediates the cancer-promoting effect of ABCC5 on prostate cancer.** A. MTS cell proliferation assay. Cell growth curves of ABCC5 and ABCC5-shCDK1 and control cells. B. Colony formation assay. Representative images of colonies of ABCC5 with or without RO-3306 treated cells (left). Quantification of the number of colonies by the described cells (right). C. Transwell migration assay. Representative images of ABCC5 and ABCC5-shCDK1 and control cells (10x, top), ABCC5 with or without RO-3306 treated cells (10x, bottom). Quantification of cell migration rate by the described cells (right). D-E. Wound healing assay. Representative images of wound-induced cell migration of ABCC5-overexpressed and CDK1-inhibited and control cells (4x, left). Quantification of migration by the described cells (right). F. ABCC5 upregulates the protein level of CDK1, which in turn promotes phosphorylation of the AR (ser81). G. ABCC5 upregulates the transcriptional activity of AR on downstream target genes. **P < 0.01. n.s., none of significance. ENZ., enzalutamide.

To verify whether CDK1 mediates the pro-oncogenic effect of ABCC5 in prostate cancer, we knocked down CDK1 again in prostate cancer cells overexpressing ABCC5 and performed a series of tumor phenotyping experiments. We found that inhibition of CDK1 expression was followed by a significant blockade of the ability of ABCC5 to proliferate and migrate in prostate cancer (Figure 6A, C, D). Then, we inhibited CDK1 kinase activity with the CDK1-specific inhibitor RO-3306 and found that the ability of highly expressed ABCC5 to promote tumor proliferation and migration was also inhibited when CDK1 kinase activity was inhibited (Figure 6B, C, D). Based on previous studies showing that CDK1 can specifically phosphorylate AR at serine 81 to activate AR transcriptional activity, we hypothesize that the pro-cancer effect of ABCC5 is mediated by stabilizing CDK1 to activate AR phosphorylation. Our experimental results also confirmed this point. After the upregulation of ABCC5 expression, not only was the protein level of CDK1 increased but also the level of AR phosphorylated at Ser81 was significantly upregulated, and the transcriptional activity of AR downstream target genes was significantly enhanced. Therefore, ABCC5 enhances the specific phosphorylation of AR at Ser81 by CDK1 through binding to CDK1 and inhibiting its protein degradation, which in turn stabilizes the protein level of CDK1 and activates the transcriptional activity of AR on downstream target genes to promote the malignant progression of prostate cancer.

### CDK1 inhibitors enhance the sensitivity of prostate cancer cells to enzalutamide

**Figure 7:**
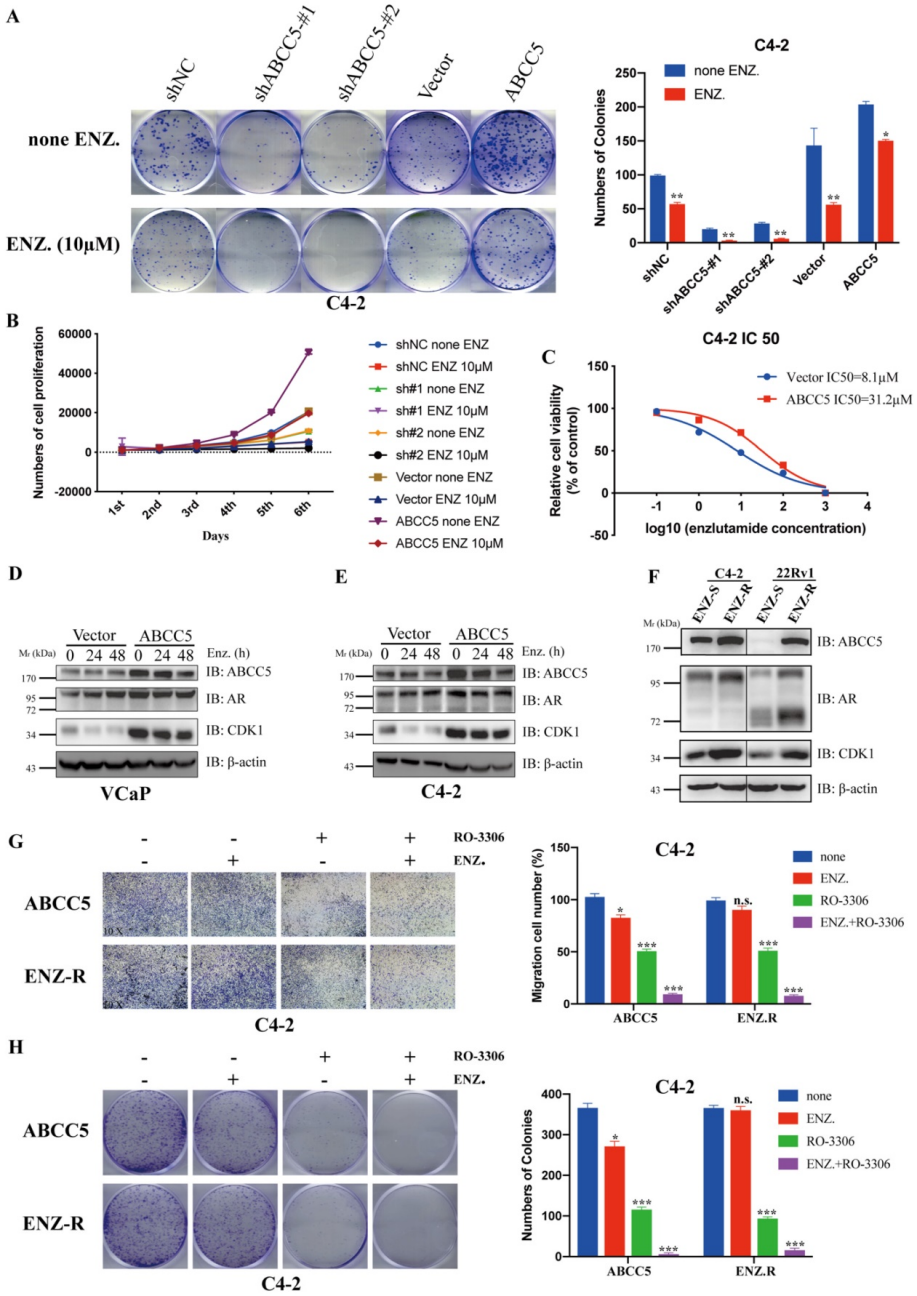
** Inhibition of CDK1 increases the sensitivity of prostate cancer cells to Enzalutamide.** A. Colony formation assay. Representative images of ABCC5-shRNA, ABCC5-overexpressed and control cells treated with or without enzalutamide (10 µM) for 24 hours (left). Quantification of colony numbers by the described cells (right). B. MTS cell proliferation assay. Cell growth curves of ABCC5-shRNA, ABCC5-overexpressed and control cells treated with or without enzalutamide (10 µM). C. Cell viability curves of ABCC5-overexpressed and control cell treated with different concentration enzalutamide (10 µM). D-E. The protein expression in ABCC5-overexpressed and control cells treated with enzalutamide (10 µM) for 0, 24, and 48 hours in VCaP (D) and C4-2 (E) cell lines. F. ABCC5 is over-expressed in ENZ-R cells. G. Transwell migration assay. Representative images of transwell migration assay of ABCC5-overexpressed and ENZ-R cells treated with or without RO-3306 (1 µM) and/or enzalutamide (10 µM) (10x, left). Quantification of cell migration by the described cells (right). H. Colony formation assay. Representative images of colony formation of ABCC5-overexpressed and ENZ-R cells treated with or without RO-3306 (1 µM) and/or enzalutamide (10 µM) (10x, left). Quantification of colonies number by the described cells (right).

ABCC5 has been highly associated with tumor cell resistance in previous studies 16, 19, 30-32. Enzalutamide resistance in prostate cancer, especially in patients with castration-resistant prostate cancer, has become an important challenge in clinical treatment. We investigated whether the mechanism of ABCC5-CDK1 in prostate cancer is related to enzalutamide resistance. First, we found that upregulated ABCC5 reduced the sensitivity of CRPC cells to enzalutamide (Figure 7A-C). We treated CRPC cells overexpressing ABCC5 with enzalutamide for a short period of time and found that enzalutamide did not significantly alter the protein expression of ABCC5, CDK1 and AR (Figure 7D-E). However, significant upregulation of ABCC5, CDK1, and AR expression was found in ENZ-R cells, suggesting that the formation of enzalutamide resistance is associated with the ABCC-CDK1 pathway. Finally, we found that the combination of the CDK1 inhibitor RO-3306 and enzalutamide significantly increased the killing effect on CRPC cells (Figure 7G-H).

## Discussion

In this study, we discovered the mechanism of ABCC5 in tumor development, namely, promoting malignant phenotypes such as proliferation and migration of prostate cancer by stabilizing CDK1 protein levels and activating ERK signaling pathways. At present, there are very few studies related to the role of ABCC5 in prostate cancer, and the conclusion that ABCC5 is highly expressed in prostate cancer and correlates with the malignancy of the tumor in the present study fills the gap in this field of research to some extent. Similarly, in 2006, Zhang et al. also found that ABCC5 expression was higher in prostate cancer than in normal paracarcinoma tissue 33. Although Karatas and his colleagues used RT-qPCR to detect nine ABC transporter proteins, including ABCC5, in prostate cancer specimens after radical prostate cancer surgery and normal prostate tissue, they found that ABCC5 was not significantly different in the tumor and normal groups and was not associated with recurrence of prostate cancer 34. However, in this study, by comparing ABCC5 mRNA expression levels in metastatic and primary prostate cancer foci from the TCGA prostate cancer database and several GEO prostate cancer databases, we showed that ABCC5 was abnormally highly expressed in prostate cancer metastases and found a higher pathological Gleason score as well as a shorter biochemical recurrence-free survival in prostate cancers with high expression of ABCC5. This cancer-promoting effect of ABCC5 was also validated in our own samples. This finding suggests that ABCC5 plays an important role in the development of prostate cancer, especially high-risk prostate cancer and that ABCC5 can serve as a prognostic biomarker for high-risk prostate cancer and provide a new target for prostate cancer diagnosis and treatment.

The existing studies on the downstream mechanisms of ABCC5 regulation in tumorigenesis and development are relatively few and mainly focus on the upstream regulatory mechanisms of ABCC5. Some scholars found that ABCC5 is the target of miRNA-210 and miRNA-516a-3p, and the deletion of these miRNAs upregulated the protein level of ABCC5, which in turn promoted tumor cell proliferation and migration, as well as the formation of chemotherapy resistance 17, 35. Our study mainly focused on exploring the downstream regulatory mechanisms of ABCC5 in prostate cancer progression. In this study, by protein profiling and immunoprecipitation techniques, ABCC5 was found to bind to CDK1 in the cytoplasm and inhibit the ubiquitination-mediated degradation of CDK1 to upregulate the protein level of CDK1, which in turn promotes the activation of AR phosphorylation by CDK1, thus exerting a protumor effect.

Many previous studies have shown that high expression of ABCC5 is closely associated with the development of drug resistance after tumor treatment 16-19, 22, 23, 30-32, 36-39. ABCC5 is not only a signaling protein in many physiological processes via pumping cGMP out of cell, but also a drug transport pump confering multitype chemotherapy resistances. However, the substrate specificity of the ABCC5 transporter is still uncertain. Some research showed that ABCC5 was not associated with drug resistance to anthracycline antibiotics, mitoxantrone, *Vinca* alkaloids, paclitaxel, and cisplatin 40, 41, while in later studies presented that ABCC5 could contribute to diminish anticancer efficacy of doxorubicin, 6-mercaptopurine (6-MP), 6-thioguanine (6-TG), 5-FU, paclitaxel, irinotecan, celecoxib, and 9-(2-phosphonylmethoxyethyl) adenine (PMEA) 42-44. In fact, our results showed that high expression of ABCC5 reduced the sensitivity of prostate cancer cells to enzalutamide. The IC50 of enzalutamide in ABCC5-overexpressing prostate cancer cells was 31.2 μM, which was significantly higher than that in the control group (Figure 7). Moreover, in enzalutamide-resistant prostate cancer cells, we found that the expression level of ABCC5 was upregulated compared to that in enzalutamide-sensitive tumor cells. This finding suggests that ABCC5 plays a vital role in the development of enzalutamide resistance in prostate cancer cells. Our previous study found that the immune microenvironment in metastatic and primary sites of prostate cancer is very different, suggesting whether ABCC5 plays a role in the tumor microenvironment. Then, we evaluated the relationship between the expression of ABCC5 and immune cell infiltration in prostate cancer based on the TCGA database (Supplementary Figure 4). The results showed that ABCC5 expression is significantly associated with the infiltration of B cells, CD4+ T cells, macrophages, neutrophils, and dendritic cells in the tumor microenvironment. This finding suggests that the role of ABCC5 in prostate cancer is of interest.

CDK1 is known to play an essential role in the management of the cell cycle and tumor development. Previous studies have demonstrated that high expression of CDK1 facilitates the poor prognosis of prostate cancer 45-48. In prostate cancer, the activation of CDK1 is linked with the development of CRPC and enzalutamide resistance via copy number loss of 17q22 45. CDK1 mediates AR Ser81 phosphorylation and stabilizes AR 49. Moreover, androgen-independent AR activity is driven by CDK1-mediated AR Ser81 phosphorylation in CRPC 47. Consistent with previous studies, our study found that ABCC5 binds to CDK1 and stably upregulates CDK1 protein levels by inhibiting its ubiquitination-mediated degradation, which in turn activates CDK1-mediated phosphorylation of AR Ser81, promoting the proliferation and invasive capacity of prostate cancer cells and the development of resistance to enzalutamide. The malignant proliferation and migration of prostate cancer caused by ABCC5 was significantly eliminated by inhibiting CDK1 activity. The combination of enzalutamide and RO-3306 significantly inhibited the growth of prostate cancer cells with high ABCC5 expression. This discovery will help provide valuable potential therapeutic targets for the clinical treatment of enzalutamide-resistant CRPC patients. Previous studies have found that in the cytoplasm, phosphorylated inactivated CDK1 is recognized and bound by 14-3-3 proteins 50. In the present study, protein profiling results also revealed that ABCC5 may be present in 14-3-3 protein binding and scored significantly lower than the binding score of ABCC5 to CDK1. This point reinforces that ABCC5 plays a critical role in stabilizing CDK1.

Our study has certain shortcomings or limitations. First, this study did not determine which specific protein is the E3 ligase that mediates CDK1 ubiquitination in prostate cancer. Second, we did not clarify the respective protein sequences in the binding regions of ABCC5 and CDK1 in this study, which we will continue in future studies. Finally, the role of CDK1 phosphorylation in regulating ABCC5-mediated CDK1 protein stability has not been evaluated. We are also conducting research to address these as yet unidentified mechanisms.

In conclusion, we found that the expression of ABCC5 was higher in prostate cancer tissue than in normal tissue and that prostate cancer patients with high ABCC5 expression had a poorer prognosis. Both *in vitro* and *in vivo* experiments confirmed that overexpression of ABCC5 promotes the malignant progression of prostate cancer. Furthermore, ABCC5 stabilizes the protein level of CDK1 by binding to and inhibiting the ubiquitin proteasomal degradation of CDK1, which in turn enhances the phosphorylation of AR Ser81 by CDK1 and promotes the transcriptional activity of AR on downstream target genes. Inhibition of CDK1 not only eliminates the cancer-promoting effect of ABCC5 in prostate cancer but also increases the sensitivity of prostate cancer cells to enzalutamide.

## Supplementary Material

Supplementary figures and tables.

## Figures and Tables

**Table 1:** Correlation of ABCC5 protein expression and clinicopathological characteristics in IUPU-PRAD

Clinicopathological features	ABCC5		*p*-value
	Low	High	
**Diagnosed age**			0.92
≤ 60	11	34	
> 60	26	78	
**Pathological T stage**			0.05
T1-T2	23	49	
T3-T4	14	63	
**Gleason score**			0.001
GS ≤ 7	37	73	
GS > 7	0	39	

IUPU-PRAD, Institute of Urology, Peking University prostate cancer.

**Table 2:** Univariable and multivariable Cox regression analyses of progression-free survival in TCGA-PRAD cohort

Characteristics	Univariable analyses		Multivariable analyses	
	HR (95% CI)	*p*-value	HR (95% CI)	*p*-value
Diagnosed age (>60 vs. ≤60)	1.413 (0.823, 2.425)	0.2	1.209 (0.687, 2.127)	0.51
Gleason Score (>7 vs. ≤7)	5.434 (2.864, 10.31)	< 0.001	3.447 (1.593, 7.459)	0.00167**
pT (T3-4 vs. T1-2)	3.428 (1.672, 7.027)	< 0.001	2.051 (1.879, 4.783)	0.034*
pN (N1 vs. N0)	1.656 (1.276, 3.133)	< 0.001	1.364 (1.046, 2.428)	0.027*
ABCC5 expression (high vs. low)	4.069 (2.18, 7.596)	< 0.001	2.281 (1.106, 4.706)	0.025*

TCGA-PRAD, The Cancer Genome Atlas Prostate Adenocarcinoma.
