# Supplementary material for: Upregulation of ATP Binding Cassette Subfamily C Member 5 facilitates Prostate Cancer progression and Enzalutamide resistance via the CDK1-mediated AR Ser81 Phosphorylation Pathway
Source: Int J Biol Sci. 2021 Apr 12;17(7):1613–28. doi: 10.7150/ijbs.59559 (PMC8120459; doi:10.7150/ijbs.59559)
Supplement: Supplementary file 1 — Supplementary figures and tables. [file ijbsv17p1613s1.pdf]

Supplementary Table S1. Characteristics of the corresponding shRNA sequences included in the context.

shRNA	Sequence (5' to 3'')
shABCC5-#1	CCGGCACCGCCAGTTGAGATCAATTCTCGAGAATTGATCTCAACTGGCGGTGTTTTTG
shABCC5-#2	CCGGTCTGTCGCCTTAGCATGTTTGCTCGAGCAAACATGCTAAGGCGACAGATTTTTG
shCDK1-#1	CCGGGGAACTTCGTCATCCAAATATCTCGAGATATTTGGATGACGAAGTTCCTTTTTG
shRNA-NC	CCGAATTACTCCTAGAACCGC

Supplementary Table S2. Characteristics of the corresponding qPCR primers included in the context.

Genes	Forward primer	Reverse primer
ABCC5	GAAGAAAGATACAACTCTGTGCTG	GGATGTAGATGCTCCTGTCAC
AR	CATTGAGCCAGGTGTAGTGTGTG	TGGAGTTGACATTGGTGAAGGAT
ALDH1	AGCCATAACAATCTCCTCTGCT	ACCGTACTCTCCCAGTTCTC
TMPRSS2	CAGGAGTGTACGGGAATGTGATGGT	GATTAGCCGTCTGCCCTCATTTGT
PSA	ACGCTGGACAGGGGGCAAAAG	GGGCAGGGCACATGGTTCACT
CDK1	TACACATGAGGTAGTAACACTCTG	AGTCCTGTAAAGATTCCACTTCTG

Supplementary Table S3. Characteristics of the corresponding antibodies included in the context

Protein	Assay	Antibody	Origin	Dilution	Incubation period
ABCC5	IHC, WB, IF	AA7906N, Invitrogen	Rabbit	1:200, 1:2000 1:200	overnight, 4°C
CDK1	IHC, WB, IF	19532-1-AP, Proteintech	Rabbit	1:100, 1:10000, 1:200	overnight, 4°C
p-AR ser 81	WB	07-1375, Millipore	Rabbit	1:2000	overnight, 4°C
AR	WB	22089-1-AP, Proteintech	Rabbit	1:2000	overnight, 4°C
p-ERK 1/2-S217/221	WB	9911T, CST	Rabbit	1:2000	overnight, 4°C
ERK1/2	WB	16443-1-AP, proteintech	Rabbit	1:2000	overnight, 4°C
β-actin	WB	TA-09, ZSGB Bio	Mouse	1:10000	overnight, 4°C

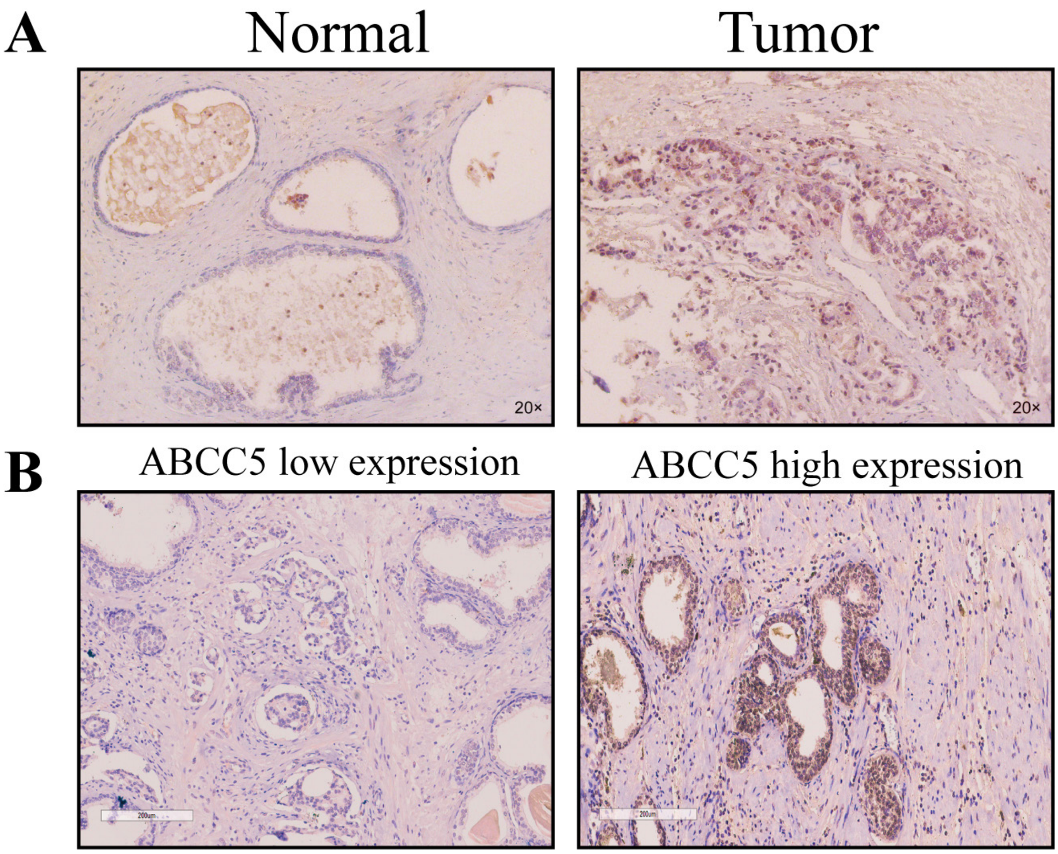

Supplementary figure 1. Representative IHC images of ABCC5 in IUPU-PRAD. IHC, immunohistochemistry; IUPU-PRAD, Institute of Urology, Peking University prostate cancer.

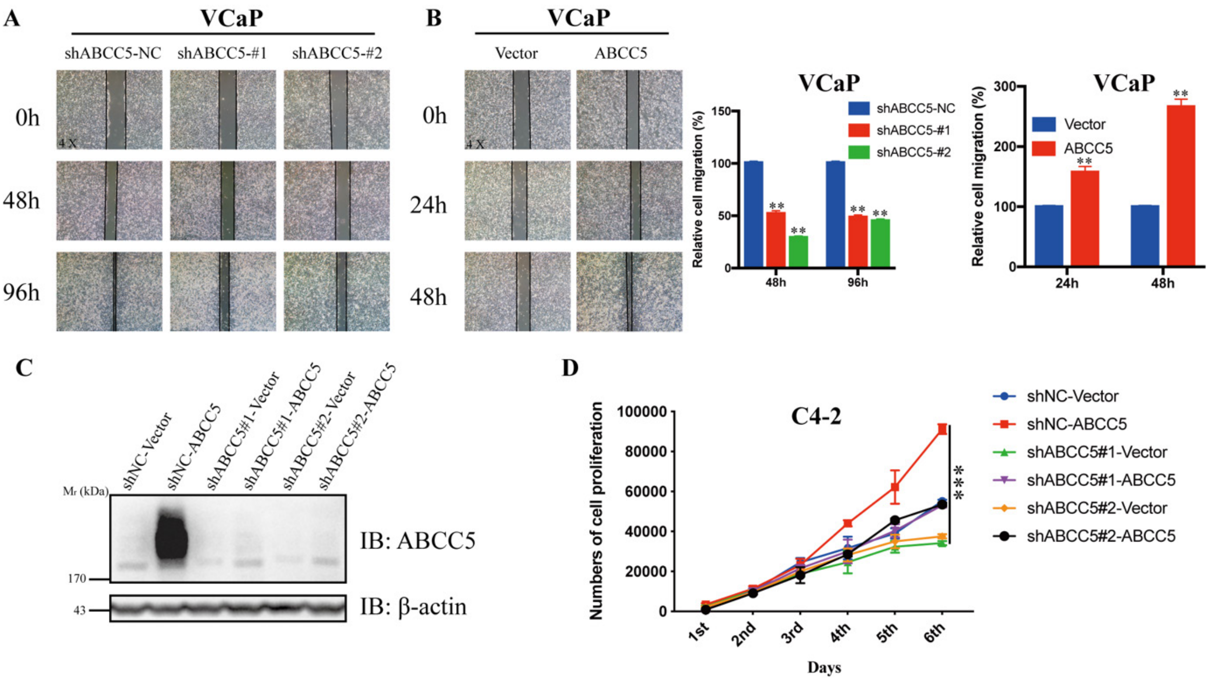

Supplementary figure 2. Rescue experiment of ABCC5 in prostate cancer. A-B. F-G. Wound healing assay, Representative images of wound-induced cell migration by the VCaP-shABCC5 (A), VCaP-ABCC5 (B) and control cells (4x, left). Quantification of migration by the described cells (right). C. ABCC5 protein expression in shABCC5-ABCC5 rescued cells and control cells. D. MTS cell proliferation assay in shABCC5-ABCC5 rescued cells and control cells.

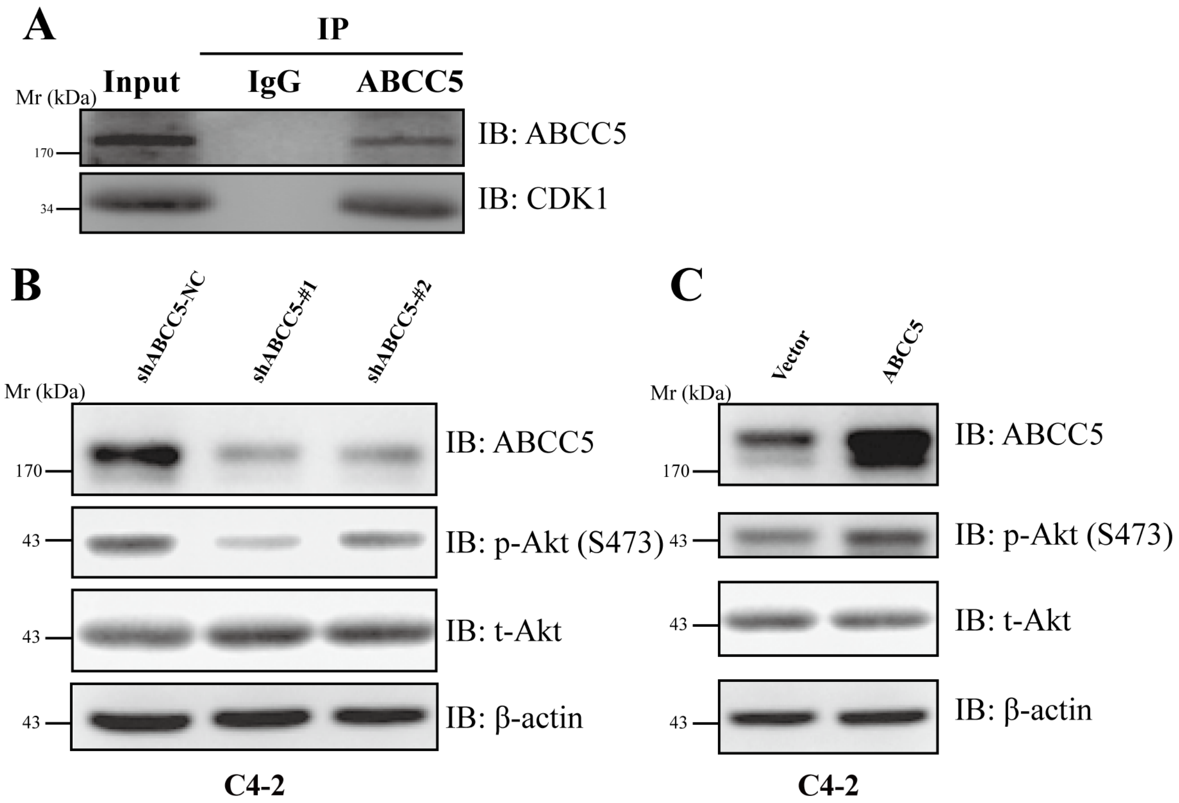

Supplementary figure 3. ABCC5 interacts with CDK1 and activates p-Akt signaling pathway. A. The result of endogenous immunoprecipitation revealed that ABCC5 binds to CDK1 at the protein level ($p < 0.01$). K-L. Knockdown and overexpression of ABCC5 can inhibit and activate the p-Akt pathway in prostate cancer cells, respectively.

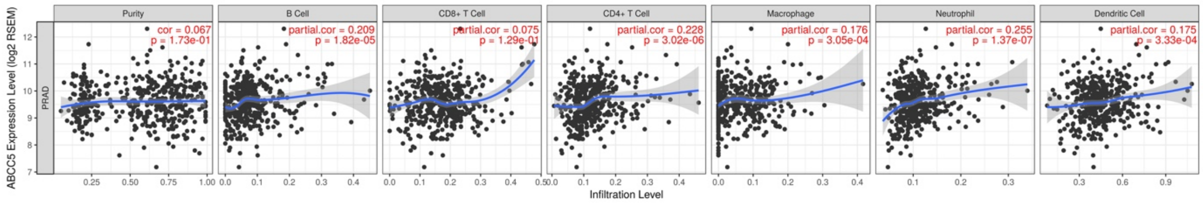

Supplementary figure 4. Correlation of ABCC5 mRNA expression and other immune cell infiltration in prostate cancer according to the TIMER algorithm.
